# Machine-learning based prediction of Cushing’s syndrome in dogs attending UK primary-care veterinary practice

**DOI:** 10.1038/s41598-021-88440-z

**Published:** 2021-04-27

**Authors:** Imogen Schofield, David C. Brodbelt, Noel Kennedy, Stijn J. M. Niessen, David B. Church, Rebecca F. Geddes, Dan G. O’Neill

**Affiliations:** 1 Pathobiology and Population Sciences, The Royal Veterinary College, Hawkshead Lane, North Mymms, Hatfield, AL9 7TA Herts UK; 2 Clinical Science and Services, The Royal Veterinary College, Hawkshead Lane, North Mymms, Hatfield, AL9 7TA Herts UK; 3 Veterinary Specialist Consultations, Loosdrechtseweg 56, 1215JX Hilversum, The Netherlands

**Keywords:** Machine learning, Animal physiology

## Abstract

Cushing’s syndrome is an endocrine disease in dogs that negatively impacts upon the quality-of-life of affected animals. Cushing’s syndrome can be a challenging diagnosis to confirm, therefore new methods to aid diagnosis are warranted. Four machine-learning algorithms were applied to predict a future diagnosis of Cushing's syndrome, using structured clinical data from the VetCompass programme in the UK. Dogs suspected of having Cushing's syndrome were included in the analysis and classified based on their final reported diagnosis within their clinical records. Demographic and clinical features available at the point of first suspicion by the attending veterinarian were included within the models. The machine-learning methods were able to classify the recorded Cushing’s syndrome diagnoses, with good predictive performance. The LASSO penalised regression model indicated the best overall performance when applied to the test set with an AUROC = 0.85 (95% CI 0.80–0.89), sensitivity = 0.71, specificity = 0.82, PPV = 0.75 and NPV = 0.78. The findings of our study indicate that machine-learning methods could predict the future diagnosis of a practicing veterinarian. New approaches using these methods could support clinical decision-making and contribute to improved diagnosis of Cushing’s syndrome in dogs.

## Introduction

Cushing’s syndrome (or hyperadrenocorticism) is an endocrine disease in dogs that occurs due to a chronic excess of circulatory glucocorticoids that ultimately produce the classical clinical signs in affected dogs^1^. Affected dogs typically show various combinations of polyuria, polydipsia, polyphagia, a potbellied appearance, muscle weakness, bilateral alopecia, panting and lethargy^1–4^. These clinical signs, along with potential consequential complications of the disease such as diabetes mellitus, pancreatitis and hypertension, highlight the importance of timely diagnosis and optimal control of Cushing’s syndrome for ongoing health and good quality-of-life^5, 6^. However, Cushing’s syndrome can be a challenging diagnosis to confirm due to non-pathognomonic clinical features making it difficult to distinguish from other possible diseases, low disease prevalence within the general dog population estimated at 0.28% and the absence of highly accurate diagnostic tests^2, 7–9^. Obtaining a correct and timely diagnosis of Cushing’s syndrome is crucial for early commencement of appropriate treatment to improve the quality-of-life of affected dogs^10^. Additionally, an incorrect diagnosis of Cushing’s syndrome could lead to unnecessary treatment which could be potentially harmful. Therefore, new methods to aid the diagnosis of Cushing’s syndrome are warranted.

A number of epidemiological studies have provided evidence for associations for several risk factors with Cushing’s syndrome such as increasing age, specific breeds and sex^2, 7^. Additionally one study has demonstrated the predictive ability of demographic and clinical features in dogs with Cushing’s syndrome under primary veterinary care in the UK, using stepwise logistic regression to develop a risk score^4^. Alternative advanced statistical and machine-learning methods are available and could offer an alternative, improved approach to standard prediction modelling^11^. Machine-learning methods have been demonstrated to outperform conventional risk models for disease prediction due to their ability to model complex, non-linear interactions between features (variables) and to handle higher numbers of features^11, 12^. Machine-learning based classification algorithms have been increasingly described in the human and veterinary medical literature, and have been applied to specific clinical problems such as using laboratory data to identify dogs with Addison’s disease and to identify cats with chronic kidney disease^11, 13–17^. A machine-learning tool to predict dogs with Cushing’s syndrome could aid veterinarians within the practice setting and could facilitate timely commencement of treatment for affected dogs.

This study aimed to explore whether novel applications of machine-learning methods to UK primary-care veterinary electronic patient records could predict a veterinarian’s recorded diagnosis of Cushing’s syndrome using clinical information at the point of first suspicion of disease.

## Results

Anonymised data were collected from 886 primary-care UK veterinary practices participating within the VetCompass programme. The study population contained 905,544 dogs attending practices in 2016, of which 10,141 were identified to have a mention of Cushing’s syndrome within their electronic patient records (EPRs). Manual revision of 62% (6287) of these EPRs identified dogs meeting the study inclusion criteria; 419 cases (recorded as having Cushing’s syndrome) and 581 non-cases (suspected of having Cushing’s syndrome but ruled out after further investigation). Dogs with no recorded information regarding clinical signs within the two-week period of first suspicion were excluded from the study, retaining 398/419 (95.0%) cases and 541/581 (93.1%) non-cases for analysis. Thirty features (variables) were extracted from the EPRs of dogs included in the study.

### Data pre-processing

Three features were removed from analysis due to near zero variance within the dataset: current administrations of insulin, l-thyroxine supplementation and anti-hypertensive agents. Six features were removed due to large proportions of missing data; body temperature (67.9% missing), heart rate (61.8%), alkaline phosphatase (ALKP) measurements (67.9%), urine specific gravity (USG) measurements (61.3%), presence of proteinuria (64.0%) and dilute USG (59.9%) at first suspicion. No high correlation between variables was identified, retaining twenty-one features (Table 1). Following one-hot encoding of breed, sex-neuter and weight change, 40 features were included in the modelling process.

**Table 1:** Descriptive statistics and univariable associations of features included in machine-learning prediction of the diagnosis of Cushing’s syndrome in dogs attending primary-care veterinary practices in the UK (Cases, n = 398; non-cases: n = 541).

Variable	Category	Cases (%)	Non-cases (%)	*p*-value
Age at first suspicion (median, IQR, years)	–	10.8 (IQR 9.0–12.5)	10.2 (IQR 8.2–12.1	0.004
Weight at first suspicion (median, IQR, kg)	–	11.4 kg (IQR 8.8–20.0)	13.2 kg (IQR 9.3–25.1)	0.008
Weight change in last 12 months (10% change)	Loss	41 (10.3)	70 (12.9)	0.41
	Gain	32 (8.0)	47 (8.7)	
	No change	325 (81.7)	424 (78.4)	
Sex-neuter	Female entire	58 (14.6)	39 (7.2)	0.001
	Female neutered	154 (38.7)	236 (43.6)	
	Male entire	53 (13.3)	61 (11.3)	
	Male neutered	133 (33.4)	205 (37.9)	
Breed	Beagle	1 (0.3)	11 (2.0)	< 0.001
	Bichon frise	32 (8.0)	24 (4.4)	
	Border collie	5 (1.3)	12 (2.2)	
	Border terrier	23 (5.8)	11 (2.0)	
	Boxer	6 (1.5)	7 (1.3)	
	Cavalier King Charles spaniel	7 (1.8)	11 (2.0)	
	Cocker spaniel	5 (1.3)	20 (3.7)	
	Crossbreed	90 (22.6)	114 (21.1)	
	Jack Russell terrier	39 (9.8)	39 (7.2)	
	Labrador retriever	6 (1.5)	39 (7.2)	
	Lhasa apso	7 (1.8)	4 (0.7)	
	Poodle	5 (1.3)	8 (1.5)	
	Schnauzer	6 (1.5)	24 (4.4)	
	Shih tzu	19 (4.8)	3 (0.6)	
	Staffordshire bull terrier	29 (7.3)	26 (4.8)	
	West Highland white terrier	13 (3.3)	46 (8.5)	
	Yorkshire terrier	20 (5.0)	20 (3.7)	
	Other purebreed	85 (21.4)	122 (22.6)	
Polydipsia	Yes	279 (70.1)	261 (48.2)	< 0.001
	No	119 (29.9)	280 (51.8)	
Polyuria	Yes	234 (58.8)	195 (36.0)	< 0.001
	No	164 (41.2)	346 (64.0)	
Polyphagia	Yes	98 (24.6)	77 (14.2)	< 0.001
	No	300 (75.4)	464 (85.8)	
Vomiting	Yes	19 (4.8)	59 (10.9)	0.001
	No	379 (95.2)	482 (89.1)	
Diarrhoea	Yes	26 (6.5)	57 (10.5)	0.03
	No	372 (93.5)	484 (89.5)	
Potbelly/hepatomegaly	Yes	197 (49.5)	116 (21.4)	< 0.001
	No	201 (50.5)	425 (78.6)	
Thin/dry skin	Yes	96 (24.1)	100 (18.5)	0.04
	No	302 (75.9)	441 (81.5)	
Alopecia	Yes	118 (29.7)	81 (15.0)	< 0.001
	No	280 (70.3)	460 (85.0)	
Pruritus	Yes	15 (3.8)	45 (8.3)	0.005
	No	383 (96.2)	496 (91.7)	
Muscle wastage	Yes	54 (13.6)	45 (8.3)	0.01
	No	344 (86.4)	496 (91.7)	
Lethargy	Yes	73 (18.3)	112 (20.7)	0.37
	No	325 (81.7)	429 (79.3)	
Panting	Yes	80 (20.1)	99 (18.3)	0.49
	No	318 (79.9)	442 (81.7)	
Neurological signs	Yes	18 (4.5)	31 (5.7)	0.41
	No	380 (95.5)	510 (94.3)	
Hospitalised in previous 12 months	Yes	55 (13.8)	81 (15.0)	0.62
	No	343 (86.2)	460 (85.0)	
Raised ALKP activity	Yes	211 (53.0)	263 (48.6)	0.001
	No	14 (3.5)	55 (10.2)	
	Unknown	173 (43.5)	223 (41.2)	
Raised ALT activity	Yes	163 (41.0)	179 (33.1)	< 0.001
	No	28 (7.0)	98 (18.1)	
	Unknown	207 (52.0)	264 (48.8)	

Data were split randomly with two-thirds of the data incorporated into a training dataset, used to optimise the prediction model (n = 626; cases = 259 and non-cases = 367). The remaining one-third of the data formed a testing dataset, used to independently evaluate the model performance (n = 313; cases = 139 and non-cases = 174).

### Model training and optimisation

Four models were trained and optimised:

(i) A Least Absolute Shrinkage and Selection Operator (LASSO) model was optimised with a penalty (lambda) of 0.014 during tenfold cross-validation. The application of this penalty term to the likelihood being maximised results in feature selection at the time of model training. The features retained in the final model included: age, specified breeds, sex, clinical signs and laboratory features (Table 2). The model demonstrated good discrimination when examining the confusion matrix during cross-validation of the training dataset with an area under the receiver operating characteristic (AUROC) curve of 0.83 (95% confidence interval (CI): 0.80–0.86) (Table 3).
(ii) A random forest (RF) model was optimised by tuning the ‘mtry’ hyperparameter to include 3 features per node split and the ‘ntree’ hyperparameter to grow 200 trees. The optimum selected threshold for the diagnosis of Cushing’s syndrome was ≥ 0.50 predicted probability which obtained a maximum PPV of 0.71 and NPV 0.72. The training dataset performance had an area under the receiver operating characteristic curve of 0.77 (95% CI 0.73–0.81). Variable importance analysis illustrated that the majority of features had small contributions to improved prediction accuracy within the RF model. Clinical signs of a potbelly and polyuria, and laboratory features (ALT and ALKP) had the greatest influence on the model.
(iii) The linear support vector machine (SVM) model was optimised using tenfold cross-validation on the training set. A range of 0.25 to 16 was searched for the cost hyperparameter and the optimal value was 0.5. The training dataset performance had an area under the receiver operating characteristic curve of 0.83 (95% CI 0.80–0.87).
(iv) The non-linear SVM model with a radial basis function (RBF) kernel was optimised by tenfold cross-validation using a grid search, tuning the cost hyperparameter to 4 (searched between 0.25 and 16) and the gamma hyperparameter to 0.02 (searched between 0.01 and 32)^18^. The training dataset performance had an area under the receiver operating characteristic curve of 0.84 (95% CI 0.81–0.87).

**Table 2:** Least Absolute Shrinkage and Selection Operator (LASSO) prediction model for a diagnosis of Cushing’s syndrome, applied to dogs attending primary-care veterinary practices in the UK (Cases, n = 259; non-cases: n = 367). Coefficients were estimated following application of a penalty (lambda = 0.014) during tenfold cross validation. Coefficients marked with a full-stop indicate coefficients that have been shrunk to zero and therefore removed from the model.

Feature	Coefficient
Age at first suspicion (years)	0.77
Weight at first suspicion (kg)	.
Beagle	−0.66
Bichon frise	0.31
Border collie	0.09
Border terrier	0.22
Boxer	.
Cavalier King Charles spaniel	0.18
Cocker spaniel	−0.11
Crossbreed	.
Jack Russell terrier	.
Labrador retriever	−1.33
Lhasa apso	0.29
Poodle	.
Other purebreed	.
Schnauzer	−0.56
Shih tzu	1.12
Staffordshire bull terrier	−0.29
West Highland white terrier	−1.17
Female neutered	.
Female entire	0.52
Male entire	.
Weight loss	−0.25
Weight gain	.
Polydipsia absent	−0.38
Polyuria present	0.64
Polyphagia present	0.06
Vomiting present	−0.46
Diarrhoea present	−0.35
Potbelly present	0.75
Thin/dry skin present	0.12
Alopecia present	0.93
Pruritus present	−0.12
Muscle wastage present	0.12
Lethargy present	.
Panting present	−0.10
Neurological signs present	.
ALKP not elevated	−1.04
ALT not elevated	−0.57
(Intercept)	−0.93

**Table 3:** Training dataset and independent test dataset performance metrics of four machine-learning models for predicting a diagnosis of Cushing’s syndrome in dogs attending primary-care practice in the UK (training dataset: cases n = 259, non-cases: n = 367; testing dataset: cases = 139, non-cases = 174; dataset prevalence = 0.44). AUROC, Area under the receiver-operating characteristic curve; PPV, positive predictive value; NPV, negative predictive value; LASSO, least absolute shrinkage and selection operator; RF, random forest; SVM, support vector machine; RBF, radial basis function.

	LASSO	RF	Linear SVM	RBF SVM
**Training dataset performance measures**				
AUROC (95% Confidence interval)	0.83 (0.80–0.86)	0.77 (0.73–0.81)	0.83 (0.80–0.87)	0.84 (0.81–0.87)
Sensitivity	0.66	0.54	0.72	0.67
Specificity	0.86	0.84	0.83	0.86
PPV	0.77	0.71	0.75	0.77
NPV	0.78	0.72	0.81	0.79
Accuracy (95% Confidence interval)	0.77 (0.74–0.81)	0.72 (0.68–0.75)	0.78 (0.75–0.82)	0.78 (0.75–0.81)
Kappa	0.53	0.40	0.55	0.54
**Testing dataset performance measures**				
AUROC (95% Confidence interval)	0.85 (0.80–0.89)	0.74 (0.68–0.79)	0.73 (0.67–0.78)	0.72 (0.66–0.78)
Sensitivity	0.71	0.49	0.63	0.58
Specificity	0.82	0.83	0.73	0.74
PPV	0.75	0.70	0.65	0.64
NPV	0.78	0.67	0.71	0.69
Accuracy (95% Confidence interval)	0.77 (0.72–0.81)	0.68 (0.63–0.73)	0.68 (0.63–0.73)	0.67 (0.61–0.72)
Kappa	0.52	0.33	0.36	0.32

The four models indicated good performance in the training dataset all with an AUROC ≥ 0.77 (Table 3). The non-linear RBF SVM model indicated the best performance during model training with the highest AUROC.

### Final model performance on test dataset

**Figure 1:**
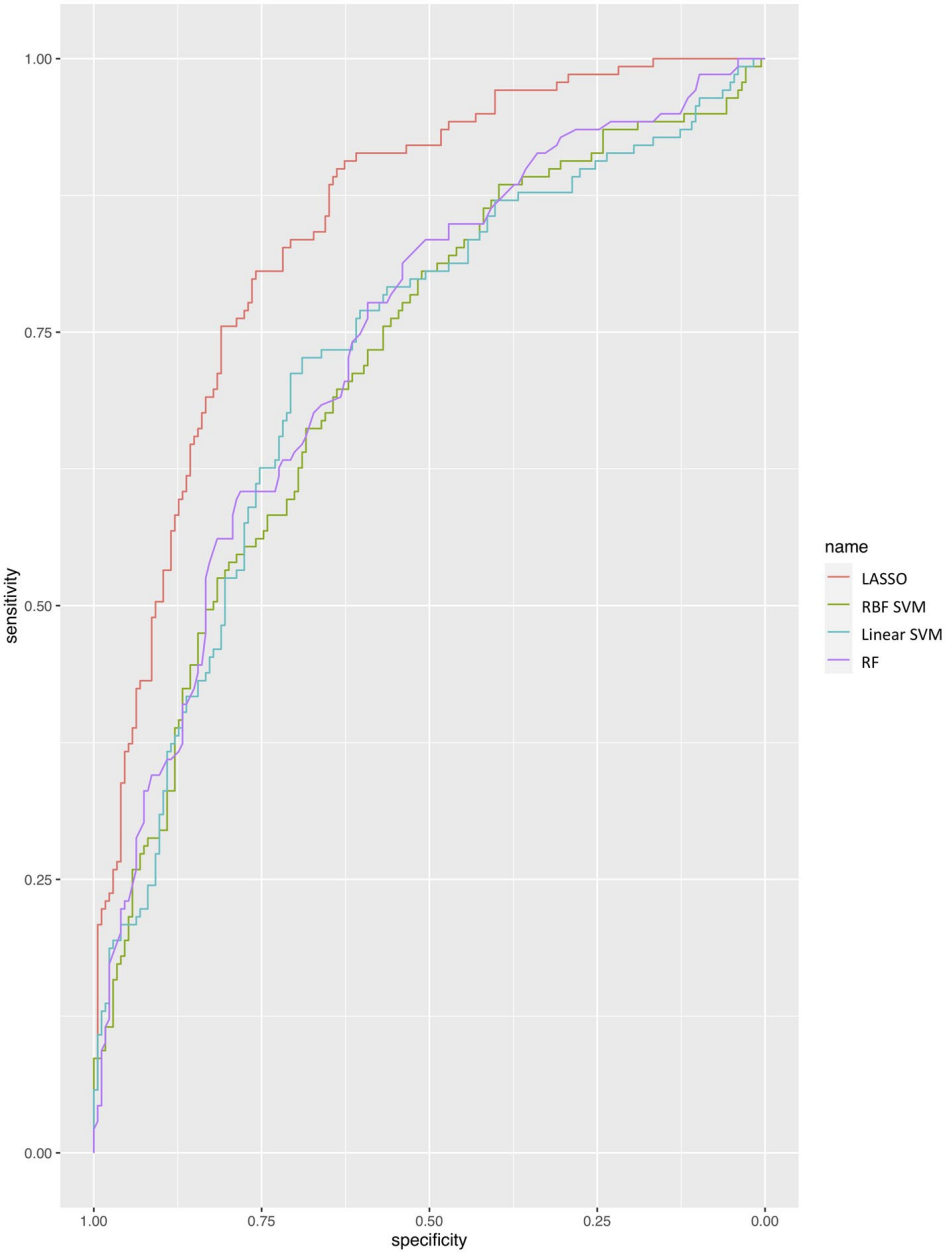
Receiver operating characteristic curve for the final prediction models for a diagnosis of Cushing’s syndrome evaluated in an independent test dataset, applied to dogs attending primary-care veterinary practices in the UK (n = 313; cases = 139 and non-cases = 174). LASSO, least absolute shrinkage and selection operator; RF, random forest; SVM, support vector machine; RBF, radial basis function.

**Figure 2:**
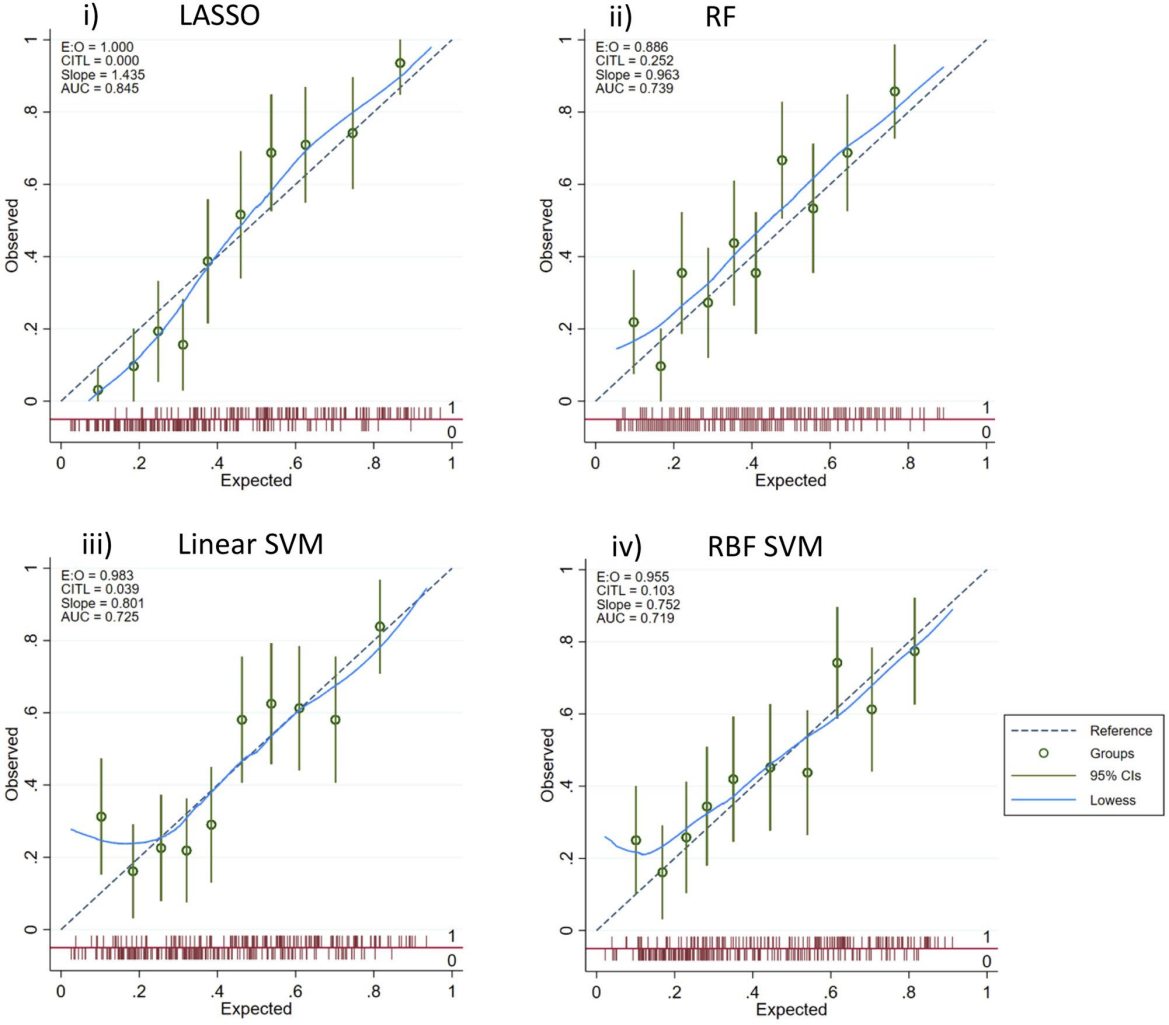
Calibration plots of the final prediction models for a diagnosis of Cushing’s syndrome, applied to dogs attending primary-care veterinary practices in the UK (n = 313; cases = 139 and non-cases = 174). The plot describes the mean observed proportions of dogs with a diagnosis of Cushing’s compared to the mean predicted probabilities, by deciles of predictions. The 45 degree line denotes perfect calibration.

Final performance of the models was assessed on the independent test dataset. All models indicated good discrimination (Fig. 1) and calibration (Fig. 2). The LASSO model indicated the best performance when applied to the test dataset (Table 4) with an AUROC = 0.85 (95% CI 0.80–0.89) which is consistent with the training cross-validation performance (Table 3). The calibration plot of the LASSO model demonstrated good model calibration with narrow 95% confidence intervals that crossed the 45 degree line of perfect calibration. The calibration plot indicates slightly poorer calibration of the lower probability predictions in the model.

**Table 4:** Confusion matrix for the Least Absolute Shrinkage and Selection Operator (LASSO) model test dataset predictions of Cushing’s syndrome in dogs attending primary-care practice in the UK (n = 313; cases = 139 and non-cases = 174).

	Observed	
Prediction	Cushing’s +ve	Cushing’s −ve
Cushing’s +ve	98	32
Cushing’s −ve	41	142

The SVM models demonstrated a drop in performance when applied to the test set: Linear SVM AUROC = 0.73 (95% CI 0.67–0.78); RBF SVM AUROC = 0.72 (95% CI 0.66–0.78) (Table 3). The RF model maintained a reasonable performance (AUROC = 0.74; 95% CI 0.68–0.79). Calibration plots suggested good calibration for the RF and SVM models (Fig. 2).

## Discussion

This study demonstrates the ability of machine-learning methods to correctly classify the recorded veterinarian diagnosis of Cushing’s syndrome in dogs from the point of first suspicion, using electronic patient records of dogs under primary veterinary care in the UK. Our study assessed four classification machine-learning models, all with good predictive performance. Of our four models, the LASSO penalised regression was the best performing model for support of a diagnosis of Cushing’s syndrome with the highest AUROC in the test set validation. The LASSO aims to selects a model that achieves a trade-off between goodness of fit and model complexity, from a large list of potential models^19^. This has been used in other prediction methods and is recommended for use in the consensus paper for medical prediction models^12, 20, 21^. Little overfitting was observed in the calibration plot of the LASSO model; however greater uncertainty was observed at the lowest predictions, with 95% confidence intervals narrowly missing the 45 degree line of perfect calibration. LASSO performs feature selection at the same time as model training, therefore requires fewer features to be considered and could be easily implemented for use in practice^22^. Another benefit of the LASSO is that it works well in low-dimensional, binary data which could be a reason for its superior performance in the classification of Cushing’s syndrome diagnosis^23^.

The RBF SVM model had the superior performance to all optimised models during the cross-validation of the training dataset with an AUROC of 0.84, however performance dropped to 0.72 in the test dataset. The RF model had the poorest performance when applied to the training datasets and retained a reasonable performance in the test dataset. There are many machine-learning methods that can be used for classification problems, each with their own advantages and drawbacks^12, 24^. A review paper examined the performance of different machine-learning methods for disease prediction and found the methods performed differently depending on the types of data used. SVM and RF models were found to perform less well than simpler models, such as regression models, when clinical and demographic data were used, which reflects the findings in the current study^24^. SVM has advantages in high dimensional datasets (considering large numbers of features) as well as for features with small predictive effects (such as for genome-wide associations)^25^. The gamma hyperparameter of the RBF SVM model affects the complexity of the model with higher values of gamma increasing the flexibility of the SVM hyperplane. In the current study, performance of the non-linear RBF SVM model was similar to the linear SVM model in the test dataset suggesting the non-linear model could have largely learnt a linear relationship. This could be due to the predominant inclusion of binary features in our dataset^22^. In our study a low gamma hyperparameter for the non-linear model was identified during model optimisation suggesting a less complex relationship was being modelled by the RBF SVM model^26^.

The drop in performance of the RBF SVM model when applied to the test dataset could indicate overfitting of the models to the training data or could be as a result of randomly splitting the data into a single training and testing group. A single train-test split is dependent on which data are randomly allocated to either group and can result in high variability between the two datasets and is less reliable at inferring generalisability of model performance^27, 28^. Other methods such as nested cross-validation can be used to reduce test set variability and could provide a less biased estimate of model generalisation performance^27, 29^. This could be used as an alternative strategy in future studies. The poorer performance of the RF model could be due to the inclusion of predominantly binary categorical variables, resulting in the model growing sparse decision trees^22, 30^. When examining the importance plot of the features in the RF model, the majority of features had a low mean decrease in accuracy and Gini which could have resulted in a model that is not highly robust. However the features with most importance were the presence of polyphagia, and polyuria as well as abnormal ALT and ALKP laboratory findings, which are clinical features frequently reported in dogs with Cushing’s syndrome^1, 9^.

Automated prediction of Cushing’s syndrome in dogs could support veterinarian decision-making and contribute to improved diagnosis of the disease. The currently available tests used for the diagnosis of Cushing’s syndrome in primary-care practice have varying sensitivities and specificities. The ACTH stimulation test is the most commonly used test in primary-care practice and has an estimated sensitivity between 57 and 83% and a specificity between 59 and 95%^2, 31–34^. The test characteristics vary according to the study referenced with superior test specificity estimates stemming from test populations which include healthy controls or controls without a clinical suspicion of Cushing’s syndrome. When applied to comparison non-case populations, similar to those used in our study, the ACTH stimulation test specificity falls between 59 and 61%^31, 32, 35^. The LDDST has an estimated sensitivity between 85 and 97% and a specificity between 70 and 73%^8, 34, 36^. In primary-care practice, these tests may be performed in dogs with a low suspicion of Cushing’s syndrome which can add to the uncertainty of interpretation for veterinarians and multiple tests are often performed, increasing the financial cost during the diagnostic process to the dog owner^4^. A prediction tool with good reliability that could be used from the point of first suspicion would offer a minimally invasive and low cost diagnostic method to support the veterinarian. Insured dogs are four times more likely to be diagnosed with Cushing’s syndrome compared to non-insured dogs, suggesting a high level of under-diagnosis related to the financial burden of gaining a diagnosis for this disease^2^. Future application of the LASSO predictive algorithm could be used to develop a computer application for mobile devices or implement it within a clinical practice management system to provide automated prediction within the consultation room^37^.

The models in this study included the information available to veterinarians during the initial stages of disease investigation; therefore these data largely include the dog’s demographic factors and presenting clinical signs. The good performance of these models suggests that discrimination of dogs with and without Cushing’s syndrome can be correctly determined at the point of first suspicion based on these factors. Due to some laboratory tests performed at external laboratories, specific measurements were therefore not routinely captured within VetCompass unless laboratory results were manually recorded within the free text clinical notes. Inclusion of specific laboratory measurement data into our study was limited. The predictive ability of these models could be improved with inclusion of additional features, with further laboratory factors offering an opportunity of future model adjustment and improved predictive performance.

There are some limitations to this study. This study used supervised machine-learning methods that require structured data for model training. In veterinary EPRs there are some standardised coding systems in place, such as VeNom coding systems, however these are not commonly used in clinical practice with the majority of information recorded as clinical free text^38^. Clinical features in this study were extracted through manual revision of the clinical notes, restricting the sample size. Future work for feature extraction using natural language processing methods or classification of clinical features could be beneficial for the clinical application of such predictive algorithms to optimise the analysis of large datasets, like VetCompass^39^. Due to the retrospective collection of the data, there is a possibility of feature misclassification and an introduction of noise, which could have diluted some predictive effects. The sample size included in this study is comparable to similar studies. However it is possible that additional training examples would support further improvements in prediction^13, 16^. Finally, further investigation on an independent dataset from a different cohort of dogs could examine the external validation of these models^40^.

In conclusion, this study applied four machine-learning models to predict the diagnosis of Cushing’s syndrome in dogs from the point of first suspicion of disease. The LASSO penalised regression model was the best performing model when applied to a held-out test dataset. The findings indicate machine-learning aided diagnosis could predict the diagnosis of a practising veterinarian and that utilising machine-learning methods as decision support tools, may contribute to improved diagnosis in Cushing’s syndrome in dogs. This study has shown that is it feasible to apply machine-learning methods to clinical data available within primary veterinary care EPRs for disease prediction and could open up the opportunities for further development in this area through application to other clinical problems.

## Methods

Data were collected from the VetCompass programme, which collates EPRs from primary-care veterinary practices in the UK. To be included in the study, dogs in the VetCompass cohort were required to have been under veterinary care in 2016 which was defined as having at least one EPR recorded during 2016 and/or at least one EPR recorded both in 2015 and 2017. To identify dogs where Cushing’s syndrome was considered as a clinical diagnosis, search terms were applied to the EPRs: ‘Cushing*, HAC, hyperadren*, hyperA, trilos*, Vetory*’. A random selection of dogs identified by the search terms were reviewed through manual revision of the EPRs. Dogs were included as a case if, (i) an initial diagnosis of Cushing’s syndrome was recorded within their EPR between 1 January 2016 and 1 June 2018 and (ii) a record was present of a low dose dexamethasone suppression test (LDDST) or adrenocorticotropic hormone (ACTH) stimulation test being performed within the EPR prior to diagnosis. Dogs were excluded as a case if a diagnosis was made prior to their first available patient record during the study period or dogs were considered to have iatrogenic Cushing’s syndrome (had glucocorticoid administration in the 30 days prior to first suspicion). Dogs were included as a comparison reference population of non-cases if, (i) there was a recorded suspicion of Cushing’s syndrome within the EPR, (ii) they subsequently had Cushing’s syndrome ruled out after undergoing a urine cortisol-creatinine ratio (UCCR) test, LDDST and/or an ACTH stimulation test between 1 January 2016 and 1 June 2018 and (iii) an alternative diagnosis was made within the EPR. Dogs with no recorded information regarding clinical signs were excluded from analysis.

Multiple features (variables) were extracted for analysis. Demographic features including breed, sex, neuter status, date of birth and bodyweight were routinely recorded within the EPRs. Breeds were categorised according to a standardised breed list adapted from the VeNom Coding Group system (Venom Coding Group 2019). Individual breeds were specified if at least 10 dogs of that breed had been included as a case or non-case. All other purebreds were grouped into a ‘purebreed other’ category. Dogs classified as a crossbreed (e.g. poodle X) or a designer breed (e.g. cockapoo) were classified into a ‘crossbreed’ category. Sex was categorised to include neuter status: female-entire, female-neuter, male-entire or male-neuter. Age at first suspicion (years) was calculated by using the date of birth and date of first suspicion of Cushing’s syndrome. Bodyweight (kg) was the bodyweight value recorded closest to the date of first suspicion. A change in weight was calculated using the recorded weight at the date of first suspicion and that recorded one year previously, where available.

Additional data were extracted manually from the EPRs. Date of first suspicion was the earliest date with evidence in the EPRs that Cushing’s syndrome was being considered as a diagnosis, and subsequently led to the veterinarian to pursue the diagnosis through further investigation. Clinical signs and routine laboratory measurements present at first suspicion (recorded one week prior and one week after the date of first suspicion) were extracted. Individual clinical signs were recorded as binary features: ‘present’ or ‘not present’ (‘not present’ was recorded if the clinical sign was specifically recorded as not present or if no information was recorded). ALKP and ALT were recorded as ‘elevated’, ‘not elevated’ or ‘unknown’ (either no test was performed or results were not reported). Proteinuria (based on a urine dipstick, including a trace recording or a urine protein-creatinine ratio) was recorded as ‘present’, ‘not present’ or ‘unknown. USG was recorded as ‘dilute’ (≤ 1.020), ‘not dilute’ (> 1.020) or ‘unknown’. Continuous data for recorded ALKP enzyme activities and USG measurements were also extracted. Treatment data (currently being received when first suspected of Cushing’s syndrome) for insulin, l-thyroxine supplementation and anti-hypertensive agents (amlodipine, benazepril, enalapril or telmisartan) were extracted^41^. Additionally clinical management data on whether dogs were hospitalised in the previous 12 months before first suspicion was included^9^.

### Data pre-processing

All analyses were performed in R version 4.0.0^42^. Features were descriptively analysed with categorical data assessed using the counts and corresponding percentages. For continuous data, normally distributed data were summarised using the mean (standard deviation (SD)) and non-normally distributed data using the median (interquartile range (IQR) and range). Variance of the features for all dogs was assessed and those with zero or near-zero variance (proportion of unique values over the sample size was < 10%) were excluded from analysis^22^. Pairwise correlations between predictor features were explored to identify collinearity using correlation coefficients; correlations (r) > 0.80 were considered highly correlated^43^. When pairs of highly correlated predictor features were identified, the variable considered to be most complete within the dataset and most clinically relevant was selected for modelling^43^. Data were assessed for missing values with features excluded if > 50% of the data was missing^44^.

The selected data were randomly split into two parts. Two-thirds (67%) of the data were allocated to a training dataset and one-third (33%) to the test dataset. Features with ≤ 50% missing data were imputed separately for training and test sets using multiple imputation by chained equations using the *mice* package in R^22, 45, 46^. Continuous variables had a normal distribution and were standardised for analysis by converting to z-scores^47^. One-hot encoding was applied to nominal features; breed, neuter-status and weight change^22^.

### Model training and optimisation

Four prediction models using different supervised machine-learning algorithms were applied to the training set: LASSO, RF, a linear SVM and a non-linear SVM. For each algorithm, hyperparameter tuning was conducted by cross-validation to optimise the models and to minimise model overfitting^48^. The hyperparameters tuned varied between the different algorithms.

(i) LASSO is a penalised regression method^49^. This method adds a penalty (lambda) to the sum of the absolute coefficients which shrinks the coefficients towards the null, with each predictor coefficient shrunk differently. The addition of a penalty reduces the likelihood of the model overfitting the data to improve prediction accuracy^22^. Lambda was optimised by tenfold cross-validation^50^. The mean lambda from the cross-validation loops was applied to the training set to determine the final model coefficients and training set performance^51^. The model was applied using the *glmnet* package in R which automatically standardises the data for the estimation of predictor effects and back transforms the final regression coefficients on the original scale^51^.
(ii) RF is an ensemble learning based classification method^22^. It uses training data to construct multiple decision-trees by bootstrap resampling and classifies unseen data using the mode of the tree output decisions^30^. These decision trees have small randomised differences in characteristics, which improves generalisation performance. Tuning of the model was performed by changing the number of decision trees grown within the ensemble (‘ntree’) and the number of features randomly sampled as candidates at each tree split (‘mtry’). Variable importance was determined for each tree within the final optimised random forest model by calculating the permutation importance index as well as measuring the decrease in node impurity^22, 52^. Importance was assessed by mean decrease accuracy, indicating the mean decrease in model accuracy due to the exclusion of that feature, and by mean decrease Gini, indicating the mean decrease in node purity achieved by each feature. The model was applied using the *rpart* package in R^53^.
(iii) SVM models map training data into a multi-dimensional space and separate the binary outcome data by a hyperplane that is maximally distant from the two outcome groups^26^. This best separating hyperplane minimises classification error and maximises geometric margin of classification. Two models were assessed: a linear and a non-linear model (with a radial basis function (RBF) kernel). The non-linear kernel SVM model can learn more complex hyperplanes than a linear SVM model^22^. Model tuning was performed for the optimal cost hyperparameter for the linear SVM model using tenfold cross-validation. The optimal cost and kernel function (gamma) hyperparameters were tuned for the non-linear RBF SVM model, using a grid search with tenfold cross-validation. The two SVM models were applied using the *e1071* package in R^18^.

Models were optimised through cross-validation by maximising the area under the receiver operating characteristic curve^11^. The optimum predictive thresholds for the LASSO and RF models were identified by maximising the PPV without a detrimental decrease in the NPV as this was deemed the most clinically valuable classification for clinicians. Once the hyperparameters had been optimised via cross-validation on the training dataset, the final model parameters were then applied to the whole training dataset^11, 21, 22^. The training performance was presented by plotting the ROC curve, calculating the AUROC curve and examining the confusion matrix (outlining sensitivity, specificity, PPV, NPV, kappa statistic and accuracy). Confidence intervals for AUROC were calculated using the DeLong method^54^ and exact binomial confidence intervals were presented for accuracy^55^. The best performing model in the training set was defined by having the highest AUROC.

### Final model performance

Performance of the final, tuned models were assessed by applying the selected prediction model to the independent test dataset. Final model performance was assessed by a confusion matrix and AUROC curves to examine the discriminatory ability of the models (distinguishes between dogs that have the outcome and those that do not)^22, 56^. Calibration of the models (the agreement between the observed outcomes and predictions) was assessed by calibration plots to assess the reliability of the probability estimates of the final models^57, 58^. The plots compared the mean observed proportions of dogs with a diagnosis of Cushing’s to the mean predicted probabilities by deciles of predictions. Perfect predictions should lie on the 45 degree line^56, 57^. The best performing model in the test set was defined as having the highest AUROC and a corresponding calibration curve indicating good calibration.

### Ethical approval

Ethical approval was granted by the Royal Veterinary College Ethics and Welfare Committee (URN SR2018-1652). All methods were performed in accordance with the relevant regulations and the ARRIVE guidelines.

## Abbreviations

CI: Confidence interval
EPR: Electronic patient record
LASSO: Least absolute shrinkage and selection operator
RF: Random forest
SVM: Support vector machine
RBF: Radial basis function
UCCR: Urine cortisol-creatinine ratio
LDDST: Low dose dexamethasone suppression test
AUROC: Area under the receiver operating characteristic curve
